# Plasma testosterone concentration is correlated with circulating immune cell abundance in transgender young people on gender-affirming hormone treatment

**DOI:** 10.3389/fimmu.2025.1608543

**Published:** 2025-07-10

**Authors:** Alice A. White, Thomas Pearce, Isabelle Coenen, Xander Bickendorf, Julia K. Moore, Penelope Strauss, Liz A. Saunders, Georgia Chaplyn, Aris Siafarikas, Ashleigh Lin, Martyn French, Christian Tjiam, Deborah Strickland, Jonatan Leffler

**Affiliations:** ^1^ Translational Immunology Team, The Kids Research Institute Australia, Perth, WA, Australia; ^2^ Medical School, University of Western Australia, Perth, WA, Australia; ^3^ School of Biomedical Sciences, University of Western Australia, Perth, WA, Australia; ^4^ Child and Adolescent Health Service, Gender Diversity Service, Perth, WA, Australia; ^5^ School of Psychiatry, University of Western Australia, Perth, WA, Australia; ^6^ Youth Mental Health Team, The Kids Research Institute Australia, Perth, WA, Australia; ^7^ School of Global and Population Health, University of Western Australia, Perth, WA, Australia; ^8^ PathWest Laboratory Medicine, Perth, WA, Australia; ^9^ Vaccine Trials Group, The Kids Research Institute Australia, Perth, WA, Australia; ^10^ Department of Clinical Immunology, PathWest Laboratory Medicine, Perth, WA, Australia

**Keywords:** flow cytometry, CyTOF, transgender, B cells, T cells, oestrogen, testosterone, GAH

## Abstract

Sex hormones, such as oestrogen and testosterone, display significant immune modulatory properties. This is highly relevant for transgender (trans) people who undergo gender-affirming hormone (GAH) treatment. However, only a limited number of studies have evaluated the immunological impact of GAH treatments, and almost none have assessed the impact in trans young people. Following recruitment to the Gender and IMmunity study (GIM) (n =100), biological samples were collected from trans young people (n = 47) including: trans males (birth-registered females taking testosterone-based GAH) and trans females (birth-registered males taking oestrogen-based GAH). All trans participants had taken GAH for at least 6 months. Samples were also collected from control individuals not taking GAH (n = 53). Immune profiles were evaluated using an 18-colour flow cytometry panel. In addition, the commercially available 37-parameter MaxPar panel was used for analysis of a subset of samples (n = 36) by mass cytometry (CyTOF). Immune cell abundance was compared across experimental groups, and correlated with plasma concentrations of oestradiol and testosterone using multiple regression models. From multiple comparisons analyses grouped by birth-registered sex, several differences were detected in the trans groups compared to control groups, in particular relating to abundance of B and T cell subsets. These differences appeared to be mainly associated with levels of plasma testosterone. The most notable differences were in trans males, who had lower numbers of CD11c^+^ B cells and higher numbers of CD4^+^ regulatory T cells (Tregs) compared to control females. Using CyTOF, further analysis of B and T cells subsets revealed the frequency of naïve B cells was higher in trans males compared to control females. This also correlated with testosterone concentration in this group. Differences in the abundance of other T cell subsets were detected in both trans males and trans females, however only a decrease in CD161^+^ T effector memory cells in trans males, compared to control females, was associated with lower testosterone levels. This cross-sectional observational study of young trans individuals suggests that testosterone treatment may have immune modulatory effects, which should be investigated further, including functional studies. While oestrogen treatment was associated with differences in some immune cells in trans females compared with controls, these were generally not associated with plasma oestradiol levels but rather with testosterone levels. Continued immunological research of young trans individuals taking GAH treatment is crucial for positive long-term health outcomes.

## 1 Introduction

Sex hormones can have significant impacts on the immune system (1). These contribute to sex-based differences in susceptibility to, and risk of comorbidities from, autoimmune and infectious diseases (2–6). Females account for 80% of people diagnosed with autoimmune conditions (3). Two autoimmune conditions of relevance are multiple sclerosis (MS) and systemic lupus erythematosus (SLE) which are rare in the pre-pubertal period (6, 7). However, increasing concentrations of oestrogen observed post-puberty are associated with a significant increase in disease incidence among females (8). Males, on the other hand, are disproportionately affected by some infectious diseases including COVID-19 (9), as well as a higher lifetime risk than females of developing some cancers, including non-reproductive cancers (10).

“Gender” is a social and cultural variable that encompasses gender roles, norms and expression. This may involve identifying as a man, woman, nonbinary or other genders (11). “Biological sex,” refers to the physiological state of being male or female, based on anatomical traits, hormones, and the genetic state of being XX (female), XY (male), or intersex chromosomal variations (11). Individuals who have a gender identity that does not align with their birth-registered sex are referred to as transgender (trans henceforth), denoted as trans male, trans female or trans nonbinary individuals (2). For example, a trans male identifies as male and was registered female at birth. Individuals whose sex at birth aligns with their gender identity are referred to as cisgender (cis), denoted as cis male or cis female (2). Trans individuals may choose to engage in medical gender-affirming hormone (GAH) treatment, such as oestrogen and testosterone to align their body with their experienced gender.

A limited number of studies have investigated the impact of GAH treatment on immunological function in trans adults. These studies predominantly focus on testosterone, reporting the impact on the function of plasmacytoid dendritic cells (pDCs) (12), interferon (IFN) production (13, 14), and regulatory T cells (Treg) (15). In addition, a trend towards elevated serum anti-nuclear antibody levels in trans females compared to both cis and trans males has also been observed (16). Although large studies on clinical outcomes related to immunological impacts are missing, multiple case studies report SLE development in trans females, associated with long-term oestrogen use (17, 18), and a retrospective hospital record study has demonstrated an increased risk of MS in trans females, when compared to the expected number of cases in a reference cohort of birth-registered males (19). Most of these case studies were of adult trans individuals, however in Australia GAH may be initiated in adolescents, presenting a population with an unmet need for scientific research. The present study evaluated how GAH may impact specific immune cell profiles in young trans people (16–23 years), as part of the Gender and Immunity (GIM) study. To achieve this, circulating immune cells were assessed using our in-house developed flow cytometry panel (20), as well as the commercially available MaxPar Immune phenotyping panel evaluated using mass cytometry (CyTOF). This analytical strategy yielded high dimensional data on peripheral blood mononuclear cell populations (PBMC) as well as short-lived granulocytes, and rare cell populations in whole blood.

## 2 Materials and methods

### 2.1 Participants and sample collection

In Western Australia, trans young people can access specialist gender-affirming care through the public health system at the Child and Adolescent Health Service Gender Diversity Service (GDS) at Perth Children’s Hospital. At the GDS, trans young people (<18 years of age) may receive medical gender-affirming treatment with puberty suppression and/or testosterone, oestrogen, and antiandrogens, where appropriate, in keeping with national and international guidelines (21–23). We recruited trans young people age 16–25 who had attended the GDS and had been taking GAH for at least 6 months. Young adult control participants aged 16 – 25 (of any gender identity, and taking no hormone treatment) were recruited by word of mouth, including siblings and friends of the trans participants, as well as researchers’ friends and family members. This study was approved by the Western Australian Child and Adolescent Health Services, Human Research Ethics Committee (#RGS01337).

We acknowledge that individuals not taking GAH may identify as a man, woman, nonbinary or other genders. In addition, not all trans individuals choose to seek medical interventions and take hormones. For our analysis, we have categorised individuals based on whether they were receiving GAH, as well as their sex registered at birth. Therefore, individuals that are not taking GAH we have denoted as “control male” or “control female,” based on their birth-registered sex. The trans groups in our cohort have been denoted as “trans male” or “trans female,” based on their birth-registered sex, gender identity, and the criterion of taking GAH at the time of sample collection (24).

After informed written consent was obtained from the participant (and from parent/guardian of participants under age 18), venous blood was collected in NaHep tubes and processed within 3 hours of collection. For the Max-Par-based experiment, a balanced subset of samples were selected, based on order of recruitment, within a nine-month time period (n = 36). For this subset, 1 mL of fresh blood was utilised for staining as detailed below. For all samples, the remaining blood was spun at 700 g for 10 min and the plasma was transferred to a separate tube, spun again at 700 g for 10 min and stored at -80 °C. The remaining plasma-depleted blood was reconstituted with an equivalent volume of PBS (Thermo Fisher Scientific, USA) and layered onto a Lymphoprep density gradient (Stemcell Technologies) for isolation of PBMC. The isolated PBMC were thoroughly washed with PBS, added to freezing media (RPMI (Thermo Fisher Scientific, USA) supplemented with 50% Hi-FCS and 10% DMSO (Sigma-Aldrich, USA)) and transferred to liquid nitrogen for long-term storage.

### 2.2 Clinical data

Data on GAH regimen, dose and duration was collected at the time of blood collection through a self-report study health questionnaire. Information on recent inflammatory events and administration of anti-inflammatory drugs was also collected. Plasma concentrations of sex hormones including oestradiol (biologically active form of oestrogen) and testosterone were measured by automated chemiluminescent immunoassays using the Architect i200SR platform (Abbott, USA). This testing was performed by PathWest Laboratory Medicine WA, a National Association of Testing Authorities-accredited clinical pathology service (**Table 1**).

**Table 1:** Clinical parameters of recruited participants.

Clinical Parameter	Control females	Control males	Trans females	Trans males	p-value
Samples included (n)	25	22	14	39	n/a
Age, mean years (range)	22.50, (19.17 – 25.42)	22.25, (16.00 – 24.50)	18.07, (16.50 – 21.25)	18.85, (16.58 – 23.33)	<0.0001
Gender-affirming hormones	n/a	n/a	Oestradiol	Testosterone undecanoate or testosterone isocaproate	n/a
Current dose hormones, mean (range)	n/a	n/a	684.82 μg , (25.00 – 4000.00)	708.11 mg, (125.00 – 1000.00)	n/a
Time taking hormones, mean months (range)	n/a	n/a	24.00, (6.00 – 48.00)	16.00, (6.00 – 48.00)	n/a
Self-reported OCP use (n)	5	n/a	n/a	n/a	n/a
Systemic oestradiol concentration, mean pmol/L (range)	140.30, (20.00 – 690.00)	67.09, (20.00 – 120.00)	161.43, (59.00 – 410.00)	138.00, 47.00 – 310.00)	<0.0001
Systemic testosterone concentration, mean nmol/L (range)	1.42, (0.60 – 9.70)	18.53, (0.90 – 35.00)	6.40, (0.50 – 21.00)	13.86, (0.40 – 68.00)	<0.0001

A total of 100 samples were analysed in this study. From our participant recruitment there was uneven sampling between experimental groups. P-values were calculated using non-parametric multiple comparisons (Wilcoxon method). n/a, not applicable; OCP, oral contraceptive pill.

### 2.3 Sample processing for flow cytometry

To evaluate individual immune cell profiles, all samples from participants in the cohort (n = 100) were stained with an 18-colour immune cell flow cytometry panel (**Supplementary Table 1**). For staining, samples were block-randomised, allowing for even distribution of experimental groups in each batch. Cryopreserved PBMC were thawed at 37 °C, and slowly resuspended in RPMI supplemented with 10% Hi-FCS. Cells were washed and resuspended in PBS, and subsequently counted for viability with Trypan blue exclusion method in a haemocytometer. For each sample, approximately 1.5 x 10^6^ PBMC were transferred to a flow cytometry tube, topped up with PBS and centrifuged at 500 g, for 5 min. The cells were resuspended in 100 μL prediluted fixable viability stain (FVS780, BD Biosciences, USA) and incubated for 10 min, protected from light. Following this, 2 mL Fluorobuffer (DPBS (Thermo Fisher Scientific, USA) supplemented with 0.5% BSA (Sigma-Aldrich, USA) and 0.05% NaN_3_) was added and incubated for 5 min. Samples were centrifuged and resuspended with 100 μL of the extracellular antibody cocktail (**Supplementary Table 1**) and incubated for 30 min at 4 °C, away from light. Samples were washed again with Fluorobuffer and fixed/permeabilised using the FoxP3 transcription buffer kit (Thermo Fisher Scientific, USA) according to manufacturer instructions. Briefly, samples were resuspended in 250 μL Fix/Perm and incubated for 30 min at 4 °C. After this, samples were washed in Perm/Wash buffer and resuspended in 100 μL of the intracellular antibody cocktail (diluted in Perm/Wash buffer) (**Supplementary Table 1**) and incubated for 30 min at 4 °C. Samples were washed with Perm/Wash buffer and resuspended in Fluorobuffer and stored at 4 °C. Acquisition was performed within 2 hours of staining completion on a BD LSR Fortessa X-20, with a maximum event rate of approximately 1000 events/s. Flow data was exported as.fcs files and analysed using FlowJo software version 10.10.0 (BD Bioscience, USA).

### 2.4 Sample processing for CyTOF

For a selected subset of individuals (**Supplementary Table 2**), fresh blood (as per above) was stained with the Maxpar Direct Immune Profiling Assay (Fluidigm, USA). For this purpose, each sample was incubated with 100 U/mL heparin (Pfizer, USA) for 20 min. Following this, 270 μL of heparin-blocked blood was used to reconstitute each lyophilised antibody panel. The Myeloid and B cell Expansion Panel 2 (Fluidigm, USA) was also added to each sample, and all samples were incubated for 10 min. Following incubation, cells were washed and fixed using the Proteomic Stabiliser 1 solution (Smart Tube Inc, USA) for 10 min. Once fixation was complete, samples were stored at -80 °C until acquisition.

Once all samples in the subset had been stained, the order of samples was block-randomised for CyTOF acquisition. The day before each CyTOF acquisition, samples were thawed at 10 °C and red blood cell (RBC) lysis was performed using the Thaw-lyse buffer (Smart Tube Inc, USA). The cells were then passed through a 35 µm cell strainer and incubated for 10 min. Cells were washed and resuspended in Thaw-lyse buffer and incubated for 10 min. The lysis process was repeated twice, until the cell pellet was white. Samples were then resuspended in 125 nM Cell-ID Intercalator-Ir (Fluidigm, USA) and incubated at 4 °C overnight. On the day of CyTOF acquisition, samples were centrifuged in the Intercalation solution then resuspended in the residual volume. Samples were washed twice in MaxPar Cell Staining Buffer (Fluidigm, USA) and resuspended in Maxpar Cell Acquisition Solution (CAS) (Fluidigm, USA) and counted. Cells were washed again as previously and resuspended at 1 x 10^6^ cells/mL, then stored at 4 °C until acquisition.

Samples were acquired on the CyTOF Helios (Fluidigm, USA) with CyTOF Software. For quality control, CST was performed on the CyTOF Helios using the Tuning solution (Fluidigm, USA) on each day of acquisition. A volume equivalent to 10% of the test volume of EQ Four Element Calibration Beads (Fluidigm, USA) was added to the sample and vortex mixed prior to acquisition. All samples were kept on ice prior to acquisition. Samples were acquired with CAS solution by wide bore injector until the last 100 μL, then resuspended with CAS solution to acquire the entire sample. Files were concatenated for each sample and normalised using the acquired beads prior to being exported as .fcs files.

### 2.5 Flow cytometry and CyTOF data analysis pipeline

For flow cytometry and CyTOF experiments, initial data pre-processing for exclusion of dead cells and beads (CyTOF only) was performed in FlowJo (**Supplementary Figures 1**, **2A**). Flow cytometry data was gated manually as per (**Supplementary Figure 2B**). Flow cytometry data and CyTOF data was loaded into in R using *FlowCore* (version 2.12.0), in R studio. FlowSOM clustering and dimension reduction was performed using the *CATALYST* package (version 1.26.0) (25). MDS plots were generated to investigate the presence of potential batch effects relating to collection date and acquisition date. Uniform Manifold Approximation and Projection (UMAP) plots were used to guide manual gating of the flow cytometry data.

For analysis of CyTOF data, FlowSOM clusters were generated using markers with stable expression, denoted as ‘type’ markers. FlowSOM meta-clusters were visualised using by UMAP and annotated (**Supplementary Figure 3**). Individual clusters were exported from R as .fcs files, and the lineage of immune cell subsets was confirmed through conventional gating in FlowJo. For B and T cell subsets, additional sub-clustering was performed following conventional gating in FlowJo (**Supplementary Figures 4**, **5**). The median fluorescence intensity (MFI) of relevant markers in B and T cell subsets was calculated in *CATALYST* and exported for further analysis (**Supplementary Figures 6**, **7A, B**).

### 2.6 Statistical analysis

The plasma concentrations of oestradiol and testosterone were compared between clinical groups using multiple non-parametric comparisons (Wilcoxon method). For cross-sectional analysis, trans groups and matched a no-hormone control group (same birth-registered sex) were compared for cell abundance, and association with hormone concentration by multiple non-parametric comparisons (Wilcoxon method). To determine specific effects of hormones, linear multiple regressions were performed for relevant cell populations, reporting t-ratios. For all statistical tests, p <0.05 was considered significant, and p values <0.1 were reported as trends. All statistical analysis was performed in JMP version 17.0 (JMP Statistical Discovery LLC,USA) and visualised using GraphPad Prism 10.3.1 (GraphPad Software, USA).

## 3 Results

### 3.1 Individuals taking GAH have stable hormone levels after 6–12 months of treatment

**Figure 1:**
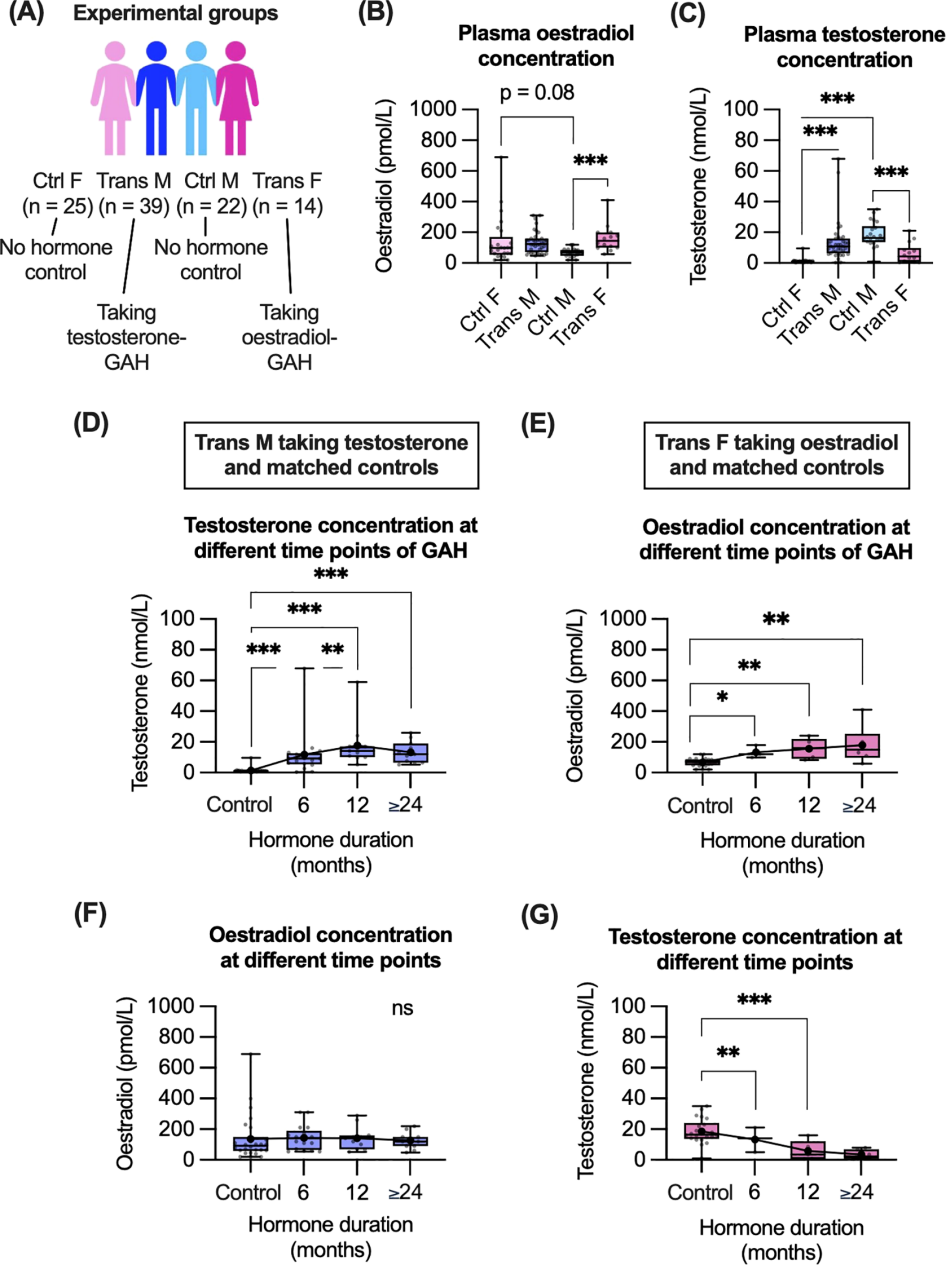
Cohort demographics and impact of GAH on plasma hormone concentrations. **(A)** Overview of participant demographics and sample sizes. **(B, C)** Plasma oestradiol **(B)** and testosterone **(C)** concentration for all groups. **(D–G)** Plasma hormone concentrations in trans males and control females **(D, F)** as well as in trans females and control males **(E, G)** based on GAH duration. Data in **(B–G)** are displayed as individual values with mean and CI indicated. Statistical significance of differences between groups was calculated using multiple non-parametric comparisons (Wilcoxon method). Significance is indicated as * p<0.05, ** p<0.01, *** p<0.001. F, female; M, male.

To evaluate the immunological impact of GAH, trans participants from the GIM study were compared to control groups who did not take hormones and were registered as the same sex at birth (**Figure 1A**). For all participants, the trans groups were on average 3–4 years younger than the controls, a statistically significant difference. Evaluating the concentration of oestradiol and testosterone (**Figures 1B, C**), levels of oestradiol were significantly higher in trans females compared to control males. There was also a trend towards higher oestradiol levels in control females compared to control males (**Figure 1B**). Testosterone concentrations were significantly higher in trans males and control males compared to control females, and were significantly lower in trans females compared to control males (**Figure 1C**).

Plasma concentration of hormones was measured at one time point for each individual. As per the cross-sectional recruitment of this study, the GAH duration varied from 0.5–4 years. To evaluate if time on GAH impacted plasma hormone levels, hormone levels were compared across time of GAH duration (**Figures 1D–G**). In trans males we observed a significant increase in testosterone concentration between 6 and 12 months of GAH treatment (**Figure 1D**). Other significant differences in hormone concentration were observed in trans groups compared to controls, however there were no additional differences in testosterone or oestradiol levels observed between 6, 12, ≥ 24 month timepoints in trans groups (**Figures 1D, E**).

Regarding endogenous hormone concentrations, in trans males there were no significant differences in oestradiol concentration for any time point (**Figure 1F**). In trans females, the endogenous testosterone concentration was only significantly decreased, compared to controls after 12 and ≥ 24 months (**Figure 1G**).

### 3.2 Both trans females and males display differential cell subset abundance compared to controls

**Figure 2:**
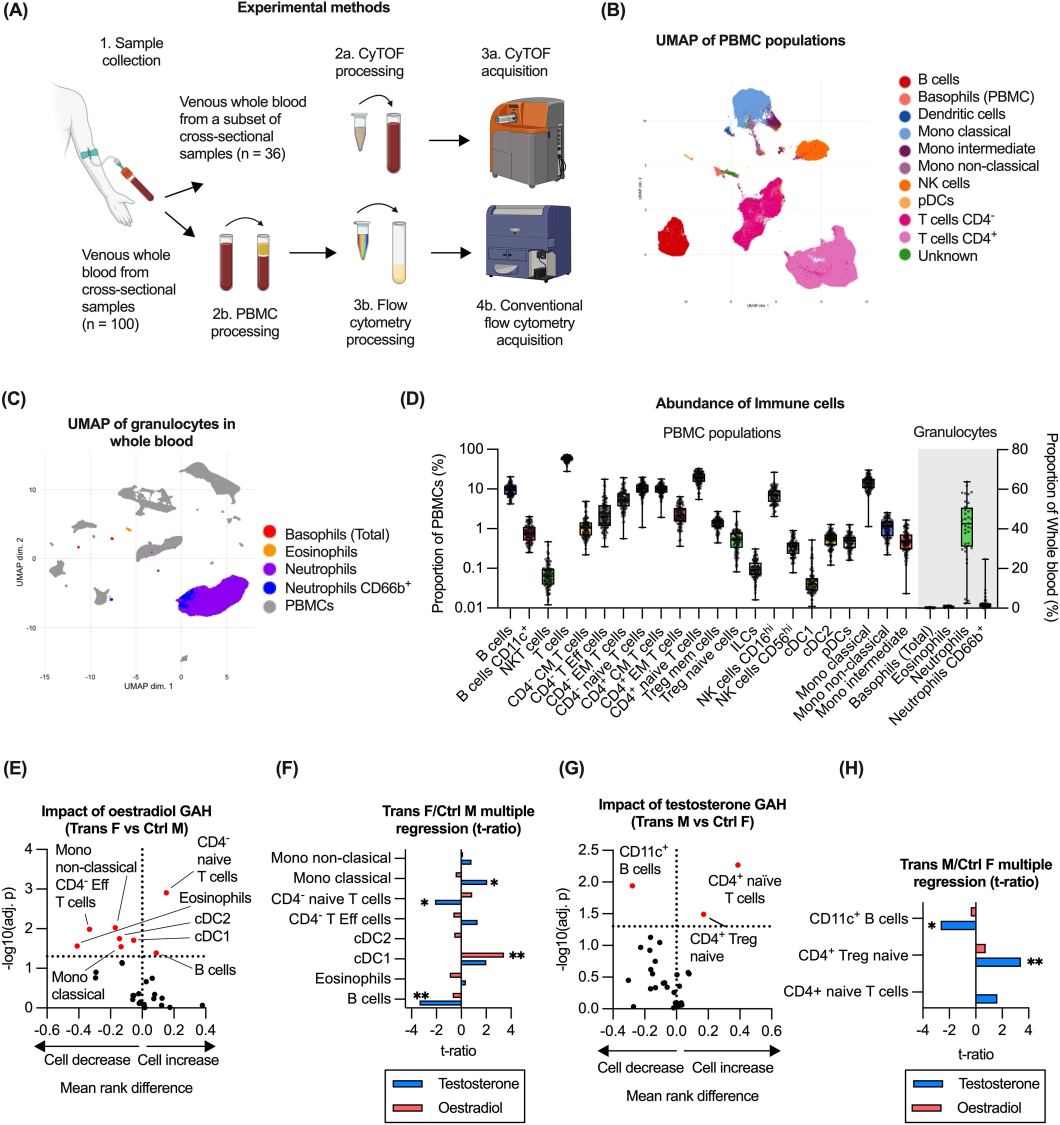
Impact of GAH on circulating immune cell populations. **(A)** Overview of experimental methods for the immune profiling methods including flow cytometry (n = 100) and CyTOF-based (n = 36) analysis. Figure created in BioRender. White, A. (2025) https://BioRender.com/wce6smv. **(B)** Identification of immune cell subsets in PBMC as measured using flow cytometry and visualised by UMAP. **(C)** Detection of granulocyte populations in whole blood as measured using CyTOF. **(D)** Quantification of immune cell subsets across flow cytometry and CyTOF data for all groups. **(E)** Comparison of immune cell abundance ratio between trans females and control males. **(F)** Multiple regression of trans female/control male groups and hormone concentration. **(G)** Comparison of immune cell abundance ratio between trans males and control females. **(H)** Multiple regression of trans male/control female groups and hormone concentration. Data in **(B–D)** is based on all available samples. In **(E, G)**, ratios between experimental groups and -log10(adj.p) values are displayed for relevant comparisons, significance of association was calculated using a multiple least-squares regression model. In **(F, H)**, the effect size from the impact of hormone concentration on each subset is displayed. Statistical significance of differences between groups was calculated using t tests. Significance is indicated as * p<0.05, ** p<0.01, *** p<0.001. CM, central memory; Eff, effector; EM, effector memory; ILC, innate lymphoid cell; Mono, monocyte; Treg, regulatory T; NK, natural killer; cDC, conventional dendritic cell; pDC, plasmacytoid dendritic cell; F, female; M, male.

To determine the impact of GAH on immune cell subsets, the abundance and phenotype of circulating immune cells was evaluated using flow cytometry (n = 100) on PBMC populations and CyTOF (n = 36) on whole blood, (**Figure 2A**). Dimension reduction was utilised to guide manual gating of flow cytometry data (**Figure 2B**, **Supplementary Figure 2B**) whereas analysis of CyTOF data was based on dimension reduction and clustering, due to the high number of parameters (**Figure 2C**). CyTOF was utilised to identify granulocyte populations not present in the PBMC preparation (**Supplementary Figure 3C**). Abundances of all immune cell subsets detected by flow cytometry or CyTOF are summarised in (**Figure 2D**). Please note, the flow cytometry panel lacked a CD8 antibody, therefore in our gating strategy CD8^+^ T cells were presumed to be present within this gate: CD45^var^/CD19^-^/CD3^+^/CD16^-^/CD56^-^/CD14^-^/CD123^-^/CD11c^-^/CD4^-^. The presumed CD8^+^ T cells are referred to as “CD4^-^” hereafter.

To evaluate the impact of oestrogen-based GAH, immune cell abundance was compared in trans females to control males (both registered males at birth). Based on this comparison, trans females displayed significantly higher proportions of both B cells and CD4^-^ (presumably CD8^+^) naïve T cells, whereas proportions of eosinophils, CD4^-^ effector T cells as well as several monocyte and dendritic cell subsets were significantly lower (**Figure 2E**). To determine if the differences were related to administered GAH or changes to endogenous hormones as a secondary effect, the association of cell abundances with plasma concentrations of both oestradiol and testosterone was evaluated using a multiple parameter regression model. From this, only a significant positive correlation of cDC1 abundance with oestradiol concentration was observed, whereas a significant negative correlation of the abundance of B cells and CD4^-^ naïve T cells with testosterone was observed. A positive correlation of testosterone with monocyte abundance was also observed (**Figure 2F**).

To assess the impact of testosterone-based GAH, immune cell subset abundance was compared in trans males and control females (both registered females at birth). Compared to control females, trans males showed significantly higher proportions of CD4^+^ naïve T cells and CD4^+^ Treg cells as well as a lower proportions of CD11c^+^ B cells (**Figure 2G**). Evaluating the association with plasma hormone levels indeed identified a significant positive correlation between testosterone levels and the abundance of CD4^+^ Tregs, and a significant negative association with the abundance of CD11c^+^ B cells (**Figure 2H**). No association with oestradiol levels was detected in this group (**Figure 2H**).

### 3.3 Testosterone contributes to expansion of naïve B cells in trans males

**Figure 3:**
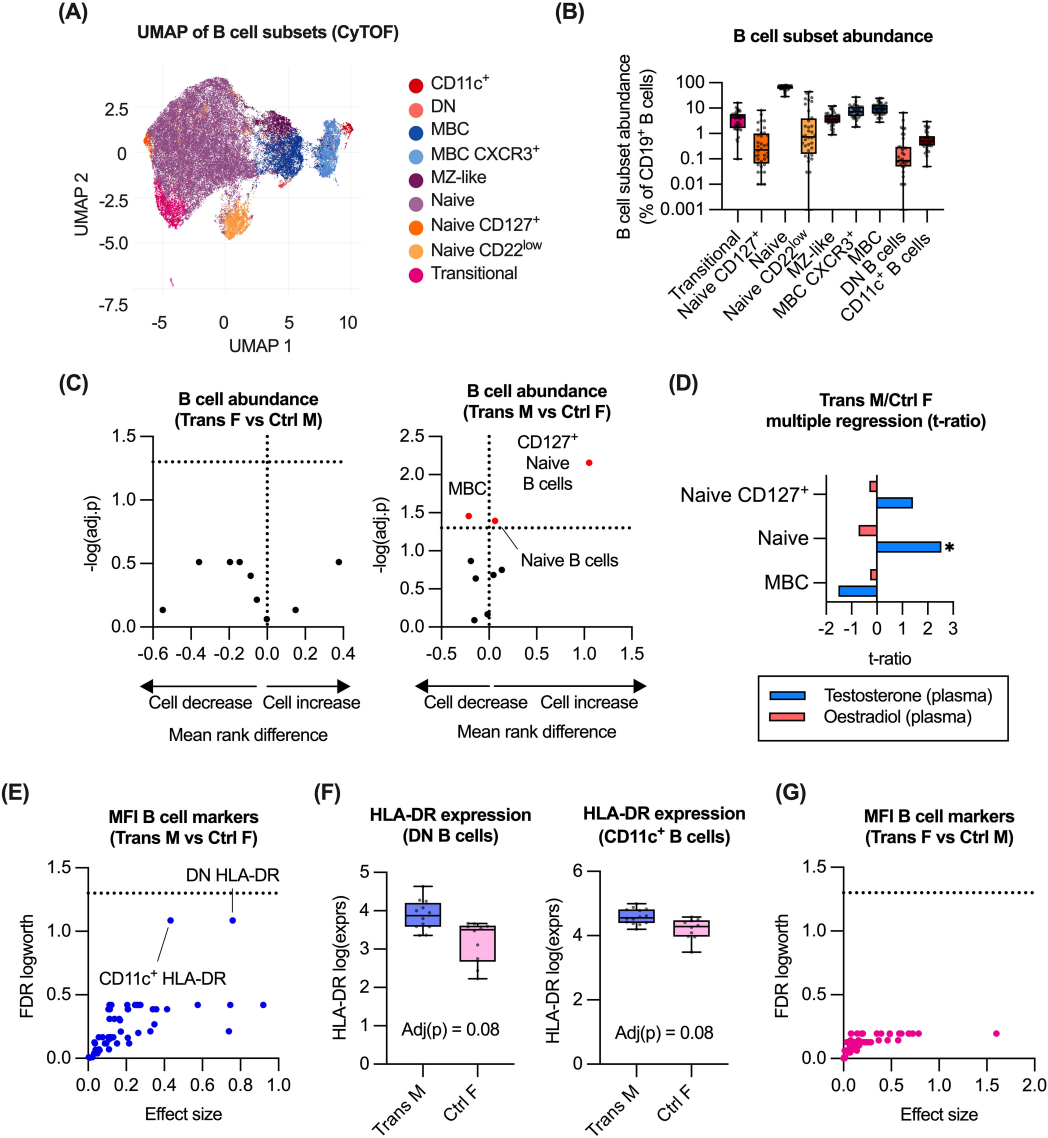
Impact of GAH on B cell subsets. **(A)** Identification of B cell subsets in CyTOF data by UMAP of manually gated total B cell populations (n = 36). **(B)** Quantification of B cell subsets as measured by CyTOF. **(C)** Comparison of B cell abundance ratio between trans females/control males, and trans males/control females. **(D)** Multiple regression of trans male/control female group and hormone concentration. **(E, G)** Response screening of p values for the mean fluorescence intensity (MFI) of relevant B cell markers. **(F)** Expression of HLA-DR on DN B cells and CD11c+ B cells was compared between trans males and control females. In **(C)**, ratios between experimental groups and -log10(adj.p) values are displayed for relevant comparisons, significance of association was calculated using a multiple least-squares regression model. In **(D)**, the effect size from the impact of hormone concentration on each subset is displayed. In **(E, G)**, response screening of p values was performed using the false discovery rate (FDR) technique. Significant or near-significant p values FDR logworth values were reported. In **(F)**, statistical significance of differences between groups was calculated using multiple non-parametric comparisons (Wilcoxon method). Significance is indicated as * p<0.05, ** p<0.01, *** p<0.001. MZ-like, marginal zone-like; MBC, memory B cell; DN, double negative; F, female; M, male.

From the flow cytometry analysis, the administration of GAH was associated with differences in the abundance of B and T cell subsets, as well as DC/monocytes. As the markers included in the immune panel provided limited resolution of B and T cell subsets, these subsets were further evaluated using the CyTOF-based data. Within the B cell population (CD45^+^/CD66b^-^/CD14^-^/CD56^-^/CD3^-^/CD19^+^), we identified nine B cell subsets, based on their expression of established B cell markers (26–28), and expression of phenotypic markers. From this analysis, the majority made up of naïve B cells (**Figures 3A, B**, **Supplementary Figure 4**).

Comparing abundance of these subsets between trans females versus control males, and trans males versus control females did not reveal any difference between trans females and control males. However, a higher proportion of naïve B cells and CD127^+^ naïve B cells was observed in trans males compared to control females (**Figure 3C**). In the same comparison, a lower proportion of memory B cells was also observed (**Figure 3C**). Evaluation of associations with plasma hormone concentrations identified a significant positive correlation between testosterone levels and naïve B cell abundance in trans males and control females (**Figure 3D**).

To assess associations between GAH and B cell function, we evaluated impact of GAH on expression (MFI) of markers associated with B cell function, such as HLA-DR, CD80, CCR7 and CD40 (**Supplementary Figure 6**). Although no significant differences in expression were observed for trans males and control females, a trend towards a testosterone-associated increase in HLA-DR expression on both double negative B cells and CD11c^+^ B cells was observed in trans males compared to control females (**Figures 3E, F**). No significant differences in expression of these markers were detected between trans females and control males (**Figure 3G**).

### 3.4 Testosterone associated with reduced abundance of CD4^+^/CD161^+^ T effector memory cells

**Figure 4:**
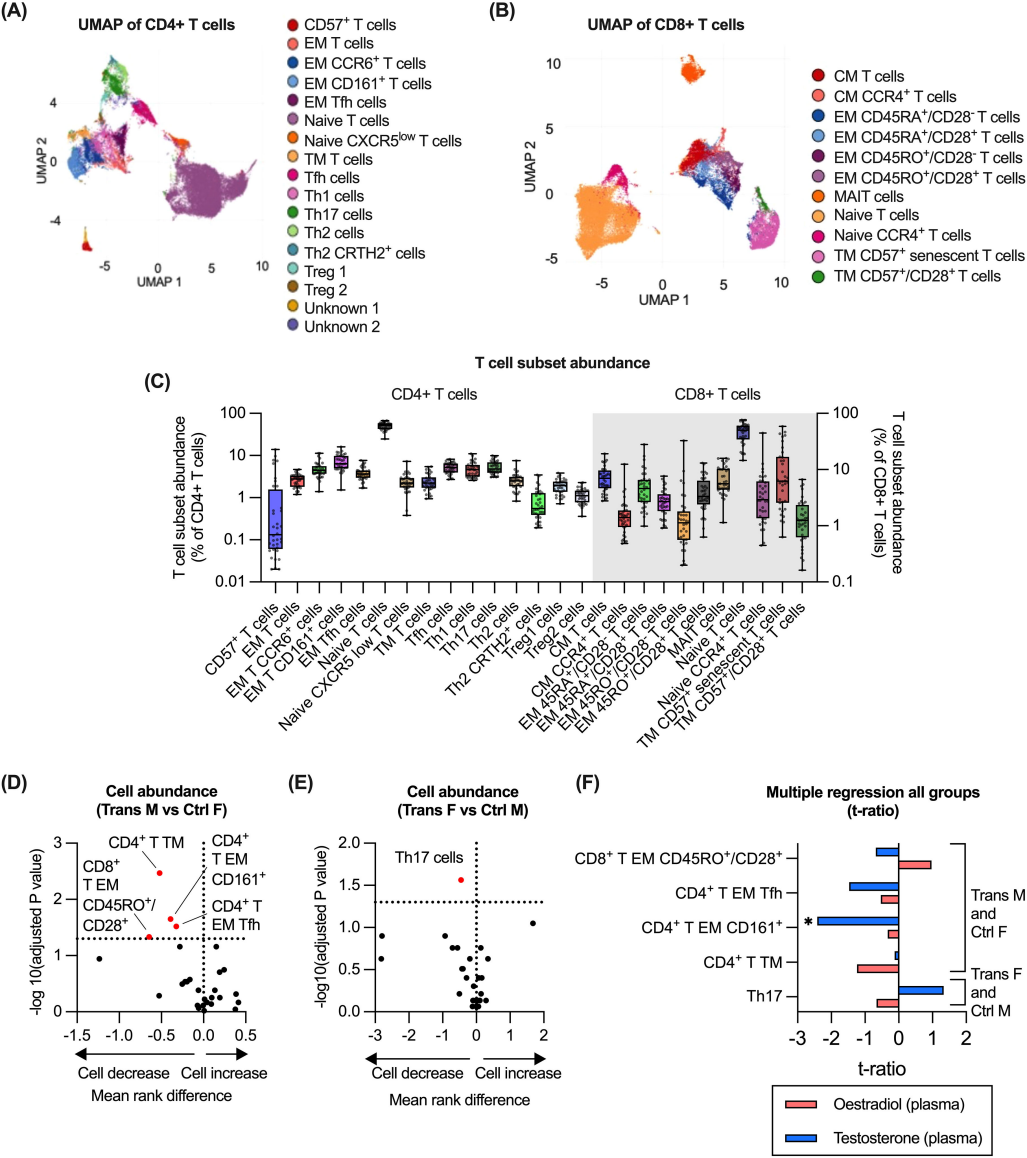
Impact of GAH on T cell subsets. **(A)** Identification of CD4+ T cell subsets in CyTOF data by UMAP of manually gated total T cell populations (n = 36). **(B)** Identification of CD8+ T cell subsets in CyTOF data by UMAP of manually gated total T cell populations (n = 36). **(C)** Quantification of all T cell subsets (CD4+ and CD8+) as measured by CyTOF cluster analysis. **(D, E)** Comparison of the abundance of T cells (CD4+ and CD8+) between trans male/control female group and trans female/control male groups. **(F)** Multiple regression of trans male/control female group and trans female/control male with hormone concentration. In **(D, E)**, ratios between experimental groups and -log10(adj.p) values are displayed for relevant comparisons, significance of association was calculated using a multiple least-squares regression model. In **(F)**, the effect size from the impact of hormone concentration on each subset is displayed. Significance is indicated as * p<0.05, ** p<0.01, *** p<0.001. EM, effector memory; Tfh, T follicular helper; CM, central memory; MAIT, mucosal-associated invariant T cells; TM, terminal memory; Treg, regulatory T; F, female; M, male.

We next evaluated the impact of GAH on T cell subsets derived from the CyTOF-based analysis. For this purpose, CD45^+^/CD66b^-^/CD14^-^/CD56^-^/CD19^-^/CD3^+^/TCRgd^-^/CD123^-^ cells were utilised to generate a total of 17 CD4^+^ and 11 CD8^+^ T cell subsets were identified (**Figures 4A, B**, **Supplementary Figure 5**). T cell populations were annotated based on their expression of established T cell markers (29–32), and expression of phenotypic markers. The relative abundance of each T cell subset was quantified (**Figure 4C**) and compared for trans males to control females, and for trans females to control males (**Figures 4D, E**). Trans males displayed significantly lower proportions of both CD4^+^ and CD8^+^ memory subsets compared to control females (**Figure 4D**). In trans females, only a lower proportion of Th17 cells compared to control males was observed (**Figure 4E**). Evaluating the association between T cell subset abundance with oestradiol and testosterone concentrations identified a significant correlation between testosterone and CD4^+^ effector memory T cells that were also positive for CD161 in trans males and control females (**Figure 4F**). Although Th17 cells were significantly lower amongst trans females compared to control males, no significant correlation with any hormone level was observed (**Figure 4F**). Finally, the association of GAH with expression of markers associated with T cell function was evaluated. These markers included CD80, CD25, CD57, CD45RA and CD45RO by CD4^+^ T cells and CD8^+^ T cells (**Supplementary Figures 7A, B**). However, no markers were differentially expressed between trans males compared to control females, or trans females compared to control males (**Supplementary Figures 7C, D**).

## 4 Discussion

In the current study, we evaluated immune cell profiles in trans males and females compared to participants with the same birth-registered sex, who were not on hormone treatment (denoted as controls). The findings suggest that testosterone treatment, in particular, was associated with several alterations to the abundance of B and T cell populations. Some of these alterations also appeared in trans females and thus may be secondary to the oestrogen-mediated impact on testosterone levels. From our measurement of sex hormones, the lack of difference in oestradiol levels between control males and control females was unexpected. This may be related to the large variation in systemic oestradiol concentrations detected in the control female group (20.00 – 690.00 pmol/L (**Table 1**)), which reflects cyclic variation, and potentially also the use of oral contraceptive pill (OCP) in this age group, although the number of control females in our study with self-reported OCP use was fewer than expected (n = 5/25) (**Table 1**).

It is important to note that as GAH treatment is individualised, there was some variation of the participants’ prescribed doses of oestradiol or testosterone within groups. The hormones testosterone and oestradiol physiologically inhibit one another’s production, and some endogenous or exogenous testosterone is physiologically metabolised to oestradiol; therefore the serum concentration of these hormones will be affected by the current and recent administration of GAH treatment. For trans females taking oral or transdermal oestrogen as in this cohort, serum levels of oestradiol are relatively stable. For trans males taking testosterone by intramuscular depot injection either 4-weekly or 3-monthly, as in this cohort, there may be large peak and trough concentration variations. We have attempted to control for variation in hormones by utilising multiple regression models to identify the strongest interaction between systemic concentration of these hormones and immune cell abundance. We posit that the variation in the prescribed doses of GAH gives this study greater external validity, as it reflects people receiving real-world health care in the local community.

B cells are antibody-producing cells with immune modulatory properties. Among adult humans, sex based differences in lymphocytes including B cells have been described in multiple ethnic groups (1). Post-puberty the numbers of B cells are higher in birth-registered females compared to males, and this trend persists throughout adulthood to old age (1). Moreover, birth-registered females generally have a higher concentration of systemic immunoglobulins, and significantly different B cell gene expression signatures in birth-registered females compared to males (1). Sex hormones have previously been shown to play an important role in the development of B cells, and oestrogen may induce B cell activation that contributes to autoimmune states (1). The findings of this study suggest that the group taking oestrogen-based GAH treatment was associated with higher B cell abundance, whereas trans males displayed increased proportion of naïve B cells and reduced memory B cells. Counterintuitively, in trans females the abundance of total B cells was negatively correlated with testosterone. Other studies have observed an inverse relationship with androgen levels and B cell abundance (33, 34), although testosterone may also impact the abundance of naïve subsets (35). The higher levels of B cells in trans females and reduced proportion of memory B cells in trans males may suggest that trans females have better antibody-mediated immunity, aligning with what is observed in birth-registered females. It may be important to determine the clinical impact of this reduction, as it is well established that birth-registered males and females display different serum concentrations of several immunoglobulins (36, 37), and this may also be impacted by GAH treatment (38). Future research should investigate the concentration of specific immunoglobulins such as vaccine antigens, to determine potential functional implications of the reduction in memory B cells in trans males.

CD11c^+^ B cells have been associated with aging, autoimmunity and chronic virus infections, particularly in birth-registered females (39, 40). We identified that the number of CD11c^+^ B cells was significantly lower in trans males compared to control females and was negatively associated with testosterone concentration. Previous findings from experimental models have suggested that oestrogen may contribute to the expansion of CD11c^+^ B cells (28, 41). Our findings suggest that testosterone may also contribute to regulating the abundance of this subset. CD11c^+^ B cells are known to accumulate in autoimmune conditions such as SLE, therefore a reduction in these cells, as observed in trans males, may be associated with protection in the context of autoimmunity. However, it may also mean that trans males have reduced immunity against chronic virus infections, malaria, or intracellular bacterial infections that are mediated by this cell type (42, 43).

From our results, the other main immune cell population that was impacted by GAH treatment was T cells. T cells are involved in cell-mediated defence against infections either directly as CD8^+^ T cells, or indirectly through CD4^+^ T helper cells. It has previously been reported that T cells exhibit significant sexual dimorphism (1). In general, birth-registered females have higher CD4^+^ T cell counts and lower CD8^+^ cell frequencies compared to birth registered males (44). In our study, we also detected changes in the abundance of T cell subsets, with higher proportions of both CD4^+^ and CD4^-^ (presumably CD8^+^) naïve T cells in trans males and females respectively, compared to control groups. CD4^-^ naïve T cells correlated with plasma levels of testosterone and there was also a trend towards a correlation for CD4^+^ naïve T cells. Androgens have been reported to mediate apoptosis of developing T cell subsets (34, 45–47). However, proportions of CD4^+^ naïve Tregs were positively correlated with testosterone concentrations. This finding aligns with an observation where medically castrated adult birth-registered males displayed low numbers and functionality of Treg populations (48). In addition, a recent study that compared Treg populations in cis and trans individuals determined that CD4^+^ Treg populations are transcriptionally sensitive to testosterone and that GAH treatment can modulate Treg function (15). As Tregs are central to reducing the risk of autoimmune or allergic disease, the positive association to androgen levels may explain the reduced risk of such diseases following puberty in birth-registered males (49–51). The clinical impact of these observations remains unclear, but the lower abundance of memory T cells may impact immune responses to specific pathogens. More research is required to interpret this finding.

Finally, we also observed several GAH-associated differences in the abundance of innate cells such as monocytes and dendritic cell subsets. Amongst trans females and control males, the abundance of classical monocytes was positively correlated with testosterone. Monocytes have previously been shown to exhibit sex-based differences in terms of abundance, and sensitivity to androgens (52). Based on this study, the concentration of testosterone in trans females declines significantly following oestrogen GAH treatment; thus observing greater abundance of monocytes in trans females is unexpected in this group. Future studies should determine whether the increased monocytes identified in trans females is associated with functional testosterone-mediated changes such as an increase in expression of TNF, CCL2 and CXCL1, as has been previously reported (53). We also identified that conventional dendritic cells (cDC1) were strongly positively correlated to oestradiol levels for trans females and control males. Conventional DC1 are responsible for cross-presentation and activating cytotoxic CD8^+^ T cells (54), however we did not observe any hormone-mediated changes in CD8^+^ T cell activation. In general, the engagement of the oestrogen receptor is known to affect DC maturation and expression of costimulatory molecules (55). However, specific effects and functional implications of oestrogen on DC subsets is not well understood.

This study has several limitations, including a small sample size, particularly in the trans female group. This is reflective of the demographics of patients seen at the GDS, and is thought to reflect the trends in age groups that trans males and females tend to present to gender services for the commencement of GAH (56, 57). Overall, our sample sizes are comparable to several other studies including trans individuals (12, 15, 58). We also experienced lower than anticipated numbers included for the CyTOF-based analysis, due to technical issues during acquisition. Nevertheless, the CyTOF data with its high number of parameters provided additional insights into the analysis of B and T cells, as well as an opportunity to evaluate the abundance of granulocyte populations that are difficult to cryopreserve and often excluded in *ex vivo* analysis on human cohorts.

In summary, although there is established evidence on the immune modulatory effects of sex hormones (1, 2), how these effects apply to trans individuals is often difficult to interpret. This study aimed to address this gap by providing a detailed analysis of immune cell profiles in trans individuals taking GAH, identifying abundances of monocytes/DCs as well as B and T cells as susceptible to GAH. Most of these changes appeared to align with known sex-based differences in immune cells, aligning with the affirmed gender. Evaluation of hormone levels revealed that not all differences were directly related to GAH, but rather associated to corresponding changes in the endogenous sex hormone concentrations. We did not observe any significant differences related to activation or phenotype of immune cells. Additional functional studies are required to validate these findings. To conclude, continued research on the immunological outcomes of GAH treatment is crucial to ensure positive long-term health outcomes for trans people (59, 60).

## Data Availability

The raw data supporting the conclusions of this article will be made available by the authors, without undue reservation.
