# Supplementary material for: Plasma testosterone concentration is correlated with circulating immune cell abundance in transgender young people on gender-affirming hormone treatment
Source: Front Immunol. 2025 Jul 10;16:1608543. doi: 10.3389/fimmu.2025.1608543 (PMC12288729; doi:10.3389/fimmu.2025.1608543)
Supplement: Supplementary file 1 [file DataSheet1.pdf]

Supplementary Material for

Plasma testosterone concentration is correlated with circulating immune cell abundance in transgender young people on gender-affirming hormone treatment

Alice A. White[1,2], Thomas Pearce[1,3], Isabelle Coenen[1], Xander Bickendorf[1,4], Julia K. Moore[4,5], Penelope Strauss[6], Liz A. Saunders[4,7], Georgia Chaplyn[4], Aris Siafarikas[1,2,4], Ashleigh Lin[7], Martyn French[2,8], Christian Tjiam[9,10], Deborah Strickland[1], Jonatan Leffler[1,*]

[1]Translational Immunology Team, The Kids Research Institute Australia, Perth, WA, Australia
[2]Medical School, University of Western Australia, Perth, WA, Australia
[3]School of Biomedical Sciences, University of Western Australia, Perth, WA, Australia
[4]Child and Adolescent Health Service, Gender Diversity Service, Perth, WA, Australia
[5]School of Psychiatry, University of Western Australia, Perth, WA, Australia
[6]Youth Mental Health Team, The Kids Research Institute Australia, Perth, WA, Australia
[7]School of Global and Population Health, University of Western Australia, Perth, WA, Australia
[8]PathWest Laboratory Medicine, Perth, WA, Australia
[9]Vaccine Trials Group, The Kids Research Institute Australia, Perth, WA, Australia
[10]Department of Clinical Immunology, PathWest Laboratory Medicine, Perth, WA, Australia

1 Supplementary Data

1.1 Supplementary Tables

SUPPLEMENTARY TABLE 1. Summary of the antibodies used in the Immune flow cytometry panel for cross-sectional analysis. Antibodies were acquired from BD and Biolegend and optimised in-house for this panel configuration. Details for dilution and clone for each antibody are as stated below.

Fluorophore	Marker	Panel	Catalog number	Antibody dilution	Clone	Vendor
BV421	CD11c	Extracellular	566877	20	BU15	BD
BV510	CD25	Extracellular	563351	20	M-A251	BD
BV605	CD1c	Extracellular	742748	20	F10/21A3	BD
BV650	CD16	Extracellular	563691	20	3G8	BD
BV711	CD197	Extracellular	566602	20	150503	BD
BV786	CD56	Extracellular	564058	20	NCAM16.2	BD
APC-Alexa 700	CD45RA	Extracellular	320113	20	HI100	Biolegend
APC-Cy7	Viability	Extracellular	565388	20	-	BD
PE-Texas Red	CD123	Extracellular	562391	20	7G3	BD
PE-Cy7	CD45	Extracellular	557748	20	HI30	BD
FITC	HLA-DR	Extracellular	564516	20	G46-6	BD
PerCP-Cy5-5	CD19	Extracellular	566396	20	SJ25C1	BD
BUV395	CD40	Extracellular	565202	20	5C3	BD
BUV496	CD4	Extracellular	612936	20	SK3	BD
BUV737	CD14	Extracellular	612763	20	M5E2	BD

BUV805	CD3	Extracellular	612893	20	SK7	BD
APC	FoxP3	Intracellular	304119	20	206D	Biolegend
PE	CD152	Intracellular	557301	5	BNI3	BD

SUPPLEMENTARY TABLE 2. Clinical parameters of participants selected for CyTOF-based evaluation.

A total subset of 36 samples were analysed by CyTOF, after exclusion for poor sample quality or low cell recovery (n = 12 samples). From our participant recruitment there was uneven sampling between experimental groups. P-values were calculated using multiple comparisons (Wilcoxon method). n/a: not applicable, ns: not significant.

Clinical parameter	Control females	Control males	Trans females	Trans males	p-value
**Samples included (n)**	8	9	4	15	n/a
**Age, mean years (range)**	22.57, (19.17 – 25.08)	22.33, (18.67 – 24.50)	18.00, (16.50 – 18.83)	18.94, (16.58 – 21.33)	***<0.0003
**Gender-affirming hormones**	n/a	n/a	Estradiol	Testosterone undecanoate or testosterone isocaproate	n/a
**Gender-affirming hormones dose, mean (range)**	n/a	n/a	88.00 μg, (50.00 – 125.00)	671.93 mg, (4.00 – 1000.00)	n/a
**Time taking hormones, mean months (range)**	n/a	n/a	30.00, (12.00 – 48.00)	21.43, (6.00 – 48.00)	n/a
**Systemic oestradiol concentration, mean pmol/L (range)**	113.89, (20.00 – 40.00)	71.00, (44.00 – 91.00)	122.25, (59.00 – 200.00)	149.27, (47.00 – 310.00)	ns
**Systemic testosterone concentration, mean nmol/L (range)**	0.96, (0.60 – 1.30)	20.13, (11.00 – 35.00)	3.45, (0.70 – 8.50)	12.71, (5.00 – 26.00)	<0.0001

1.2 Supplementary Figures

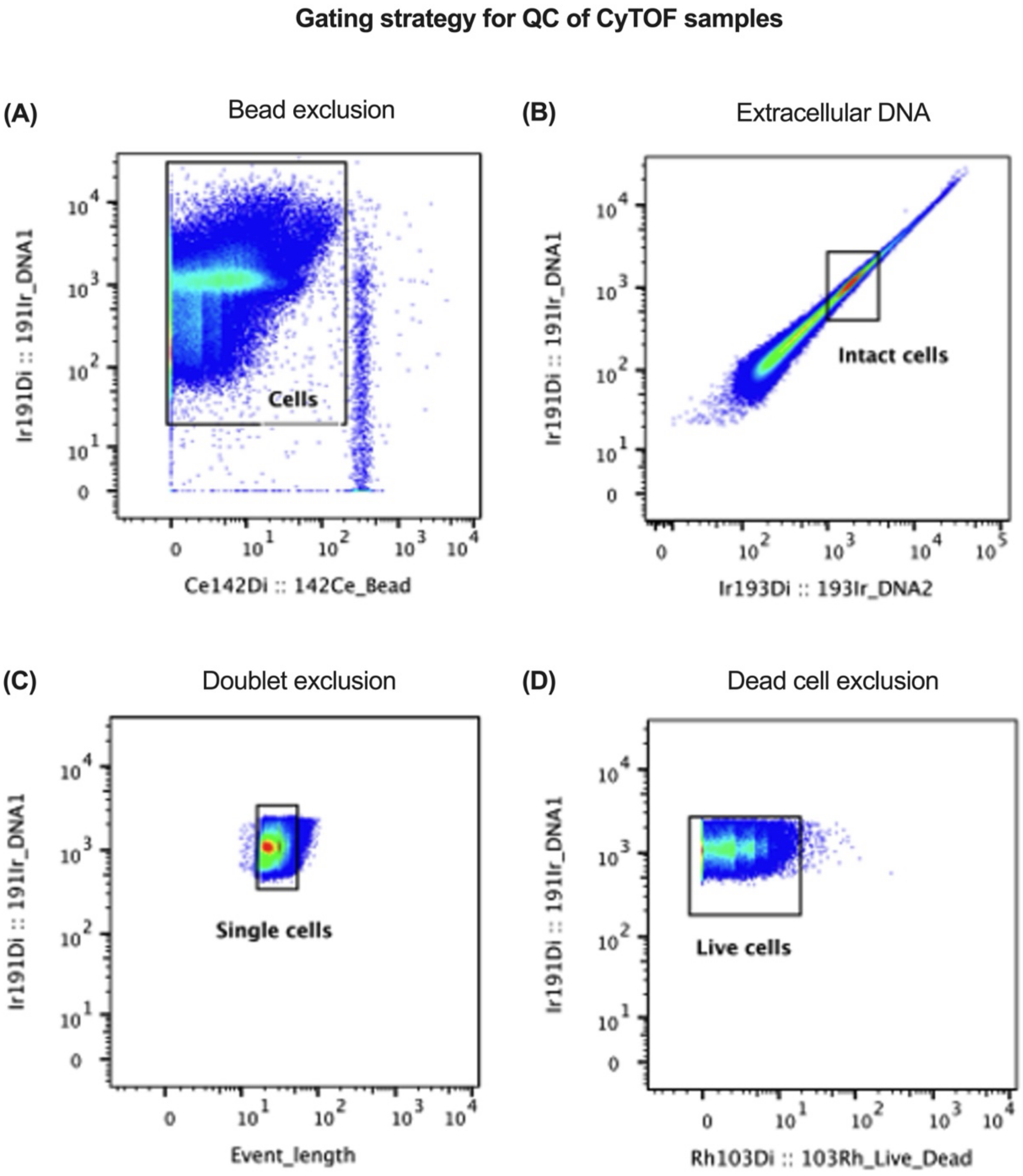

SUPPLEMENTARY FIGURE 1. Data pre-processing in FlowJo and exclusionary criteria for CyTOF data. All files were imported into FlowJo (version 10.6.2) for preliminary gating and identifying live single cells. The above quality control gating strategy excluded: **(A)** Bead populations, **(B)** Extracellular DNA, **(C)** Doublets, and **(D)** Dead cells. All samples with <50 000 total events (n=7) were excluded from the dataset. Populations from the resulting live cell gate were imported into R for cluster analysis, and gated B and T cell subsets were used for sub-clustering analysis.

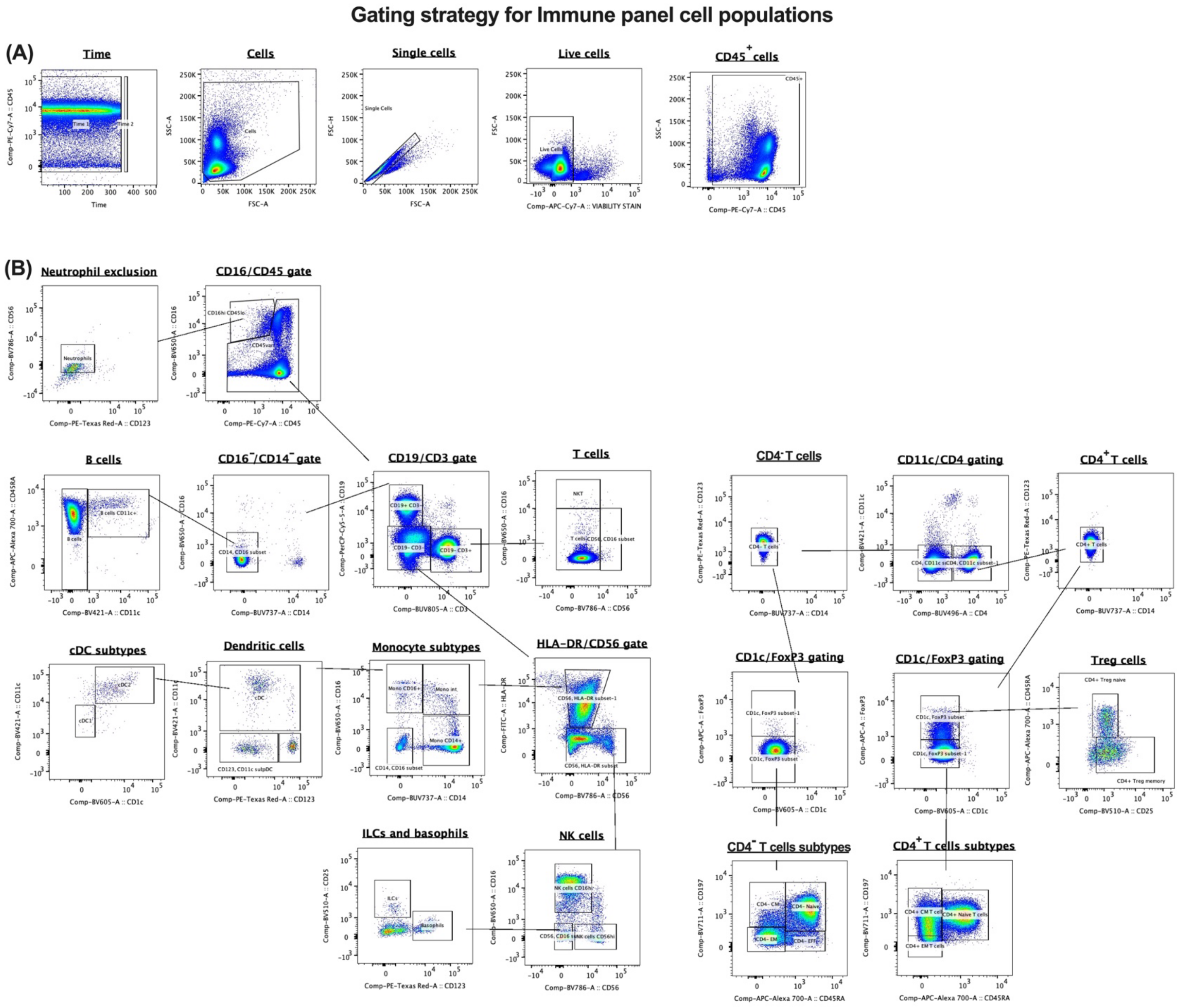

SUPPLEMENTARY FIGURE 2. Immune panel gating strategy. (A) Quality control gates were used to exclude outliers, doublets, dead cells and CD45$^-$ cells. **(B)** The cell populations of interest were isolated using the above gating strategy. Gated immune cell populations were exported for statistical analysis. cDC = conventional dendritic cell, Treg cells = regulatory T, ILCs = innate lymphoid cell, NK cell = natural killer.

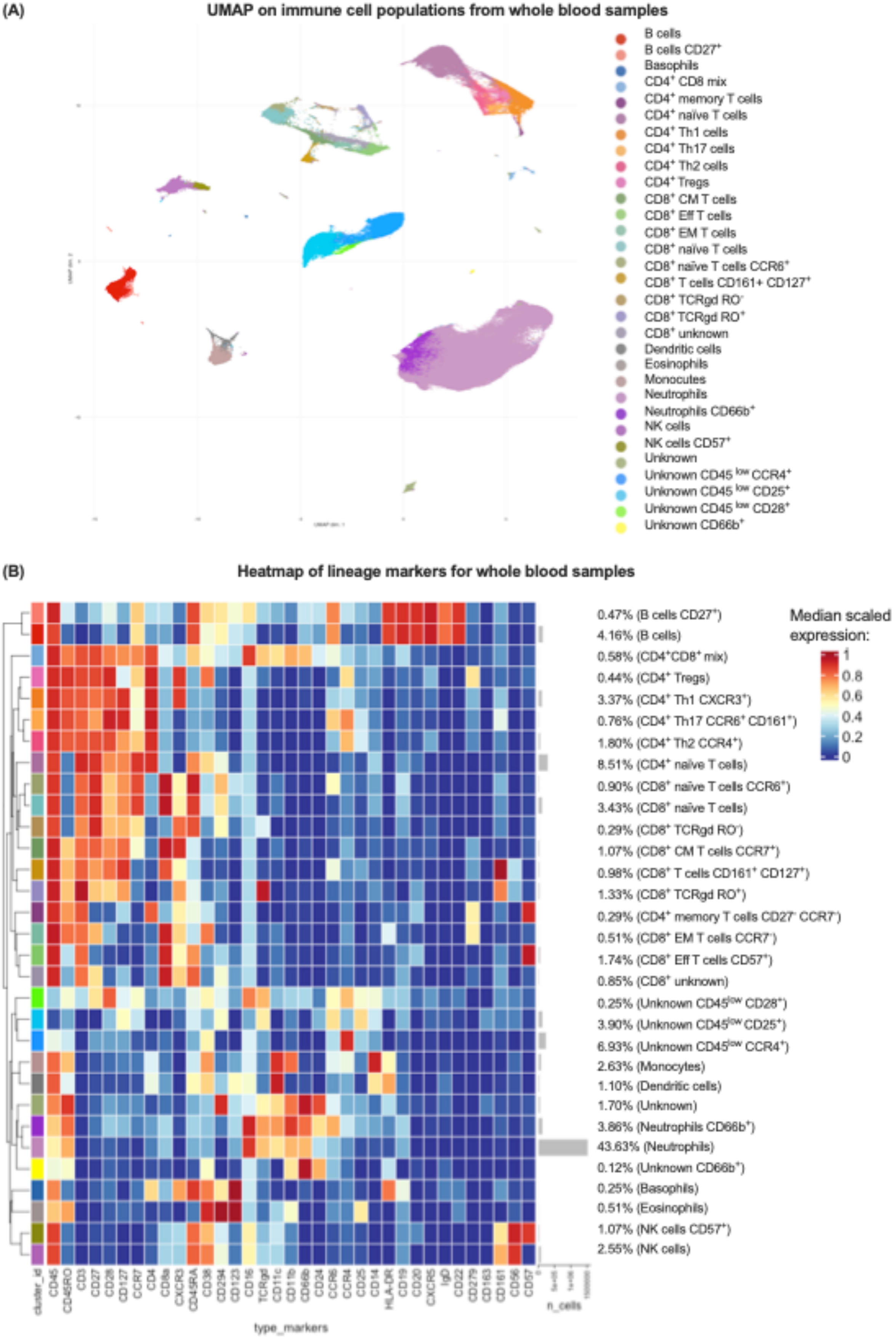
(A)
UMAP on immune cell populations from whole blood samples
B cells
B cells CD27+
Basophils
CD4+ CD8 mix
CD4+ memory T cells
CD4+ naïve T cells
CD4+ Th1 cells
CD4+ Th17 cells
CD4+ Th2 cells
CD4+ Tregs
CD8+ CM T cells
CD8+ Eff T cells
CD8+ EM T cells
CD8+ naïve T cells
CD8+ naïve T cells CCR6+
CD8+ T cells CD161+ CD127+
CD8+ TCRgd RO-
CD8+ TCRgd RO+
CD8+ unknown
Dendritic cells
Eosinophils
Monocutes
Neutrophils
Neutrophils CD66b+
NK cells
NK cells CD57+
Unknown
Unknown CD45 low CCR4+
Unknown CD45 low CD25+
Unknown CD45 low CD28+
Unknown CD66b+
(B)
Heatmap of lineage markers for whole blood samples
0.47% (B cells CD27+)
4.16% (B cells)
0.58% (CD4+CD8+ mix)
0.44% (CD4+ Tregs)
3.37% (CD4+ Th1 CXCR3+)
0.76% (CD4+ Th17 CCR6+ CD161+)
1.80% (CD4+ Th2 CCR4+)
8.51% (CD4+ naïve T cells)
0.90% (CD8+ naïve T cells CCR6+)
3.43% (CD8+ naïve T cells)
0.29% (CD8+ TCRgd RO-)
1.07% (CD8+ CM T cells CCR7+)
0.98% (CD8+ T cells CD161+ CD127+)
1.33% (CD8+ TCRgd RO+)
0.29% (CD4+ memory T cells CD27- CCR7-)
0.51% (CD8+ EM T cells CCR7-)
1.74% (CD8+ Eff T cells CD57+)
0.85% (CD8+ unknown)
0.25% (Unknown CD45low CD28+)
3.90% (Unknown CD45low CD25+)
6.93% (Unknown CD45low CCR4+)
2.63% (Monocytes)
1.10% (Dendritic cells)
1.70% (Unknown)
3.86% (Neutrophils CD66b+)
43.63% (Neutrophils)
0.12% (Unknown CD66b+)
0.25% (Basophils)
0.51% (Eosinophils)
1.07% (NK cells CD57+)
2.55% (NK cells)
Median scaled expression:
1
0.8
0.6
0.4
0.2
0
cluster_id
CD45
CD45RO
CD3
CD27
CD28
CD127
CCR7
CD4
CD8a
CXCR3
CD45RA
CD38
CD294
CD123
CD16
TCRgd
CD11c
CD11b
CD66b
CD24
CCR6
CCR4
CD25
CD14
HLA-DR
CD19
CD20
CXCR5
IgD
CD22
CD279
CD163
CD161
CD56
CD57
n_cells
type_markers

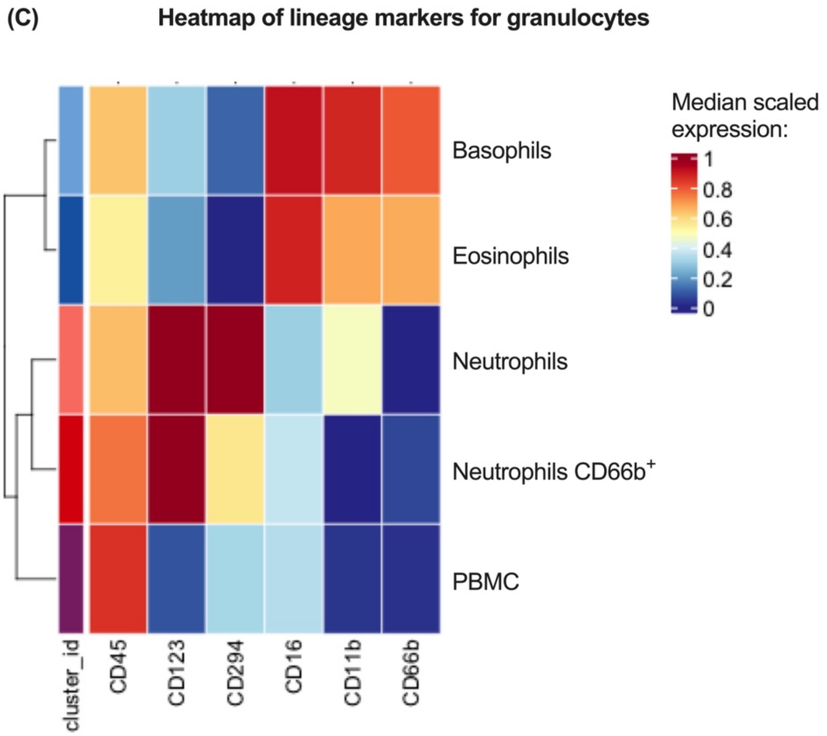

SUPPLEMENTARY FIGURE 3. Identification of Immune cell subsets in CyTOF. Whole blood was acquired by CyTOF for identification and quantification of immune cell subsets including granulocytes on all available samples (n = 36). Whole blood samples were pre-gated in FlowJo for quality control, this analysis contains cells from this lineage: Cells/Intact cells/Single cells/Live cells. **(A)** Cluster analysis of whole blood immune cells from CyTOF data by UMAP. **(B)** Heatmap of lineage markers (type markers) used to verify the identity of immune cell subsets from clustering. Cell populations are reported as percentage of total cells from the UMAP. **(C)** Heatmap of lineage markers used to verify the identity of granulocytes from clustering of whole blood populations. Tregs = regulatory T, CM = central memory, EFF = effector, EM = effector memory, NK = natural killer.

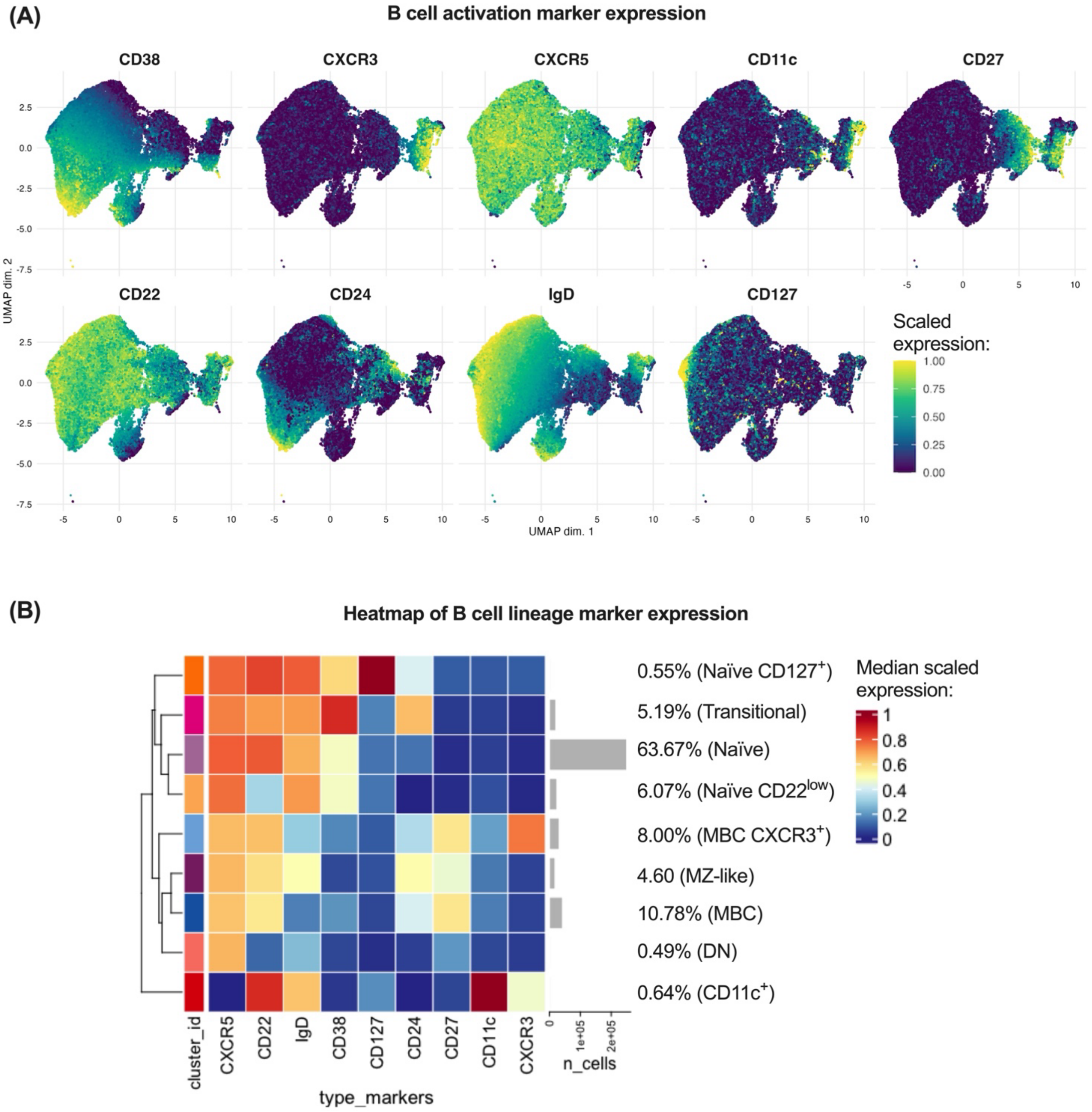

SUPPLEMENTARY FIGURE 4. Expression of state and type markers for identification of B cell subsets. Whole blood samples were pre-gated in FlowJo for quality control (n = 36), this analysis contains cells from this lineage: Cells/Intact cells/Single cells/Live cells/$CD45^{+}CD66b^{-}$/$CD14^{-}CD56^{-}$/$CD19^{+}CD3^{-}$/. **(A)** Expression of state markers (variable) on B cell subsets, displayed by UMAP **(B)** Expression of type markers (stable) on B cell subsets displayed by heatmap. Cell populations are reported as percentage of total B cells from the UMAP. MBC = memory B cell, MZ-like = marginal zone-like, DN = double negative.

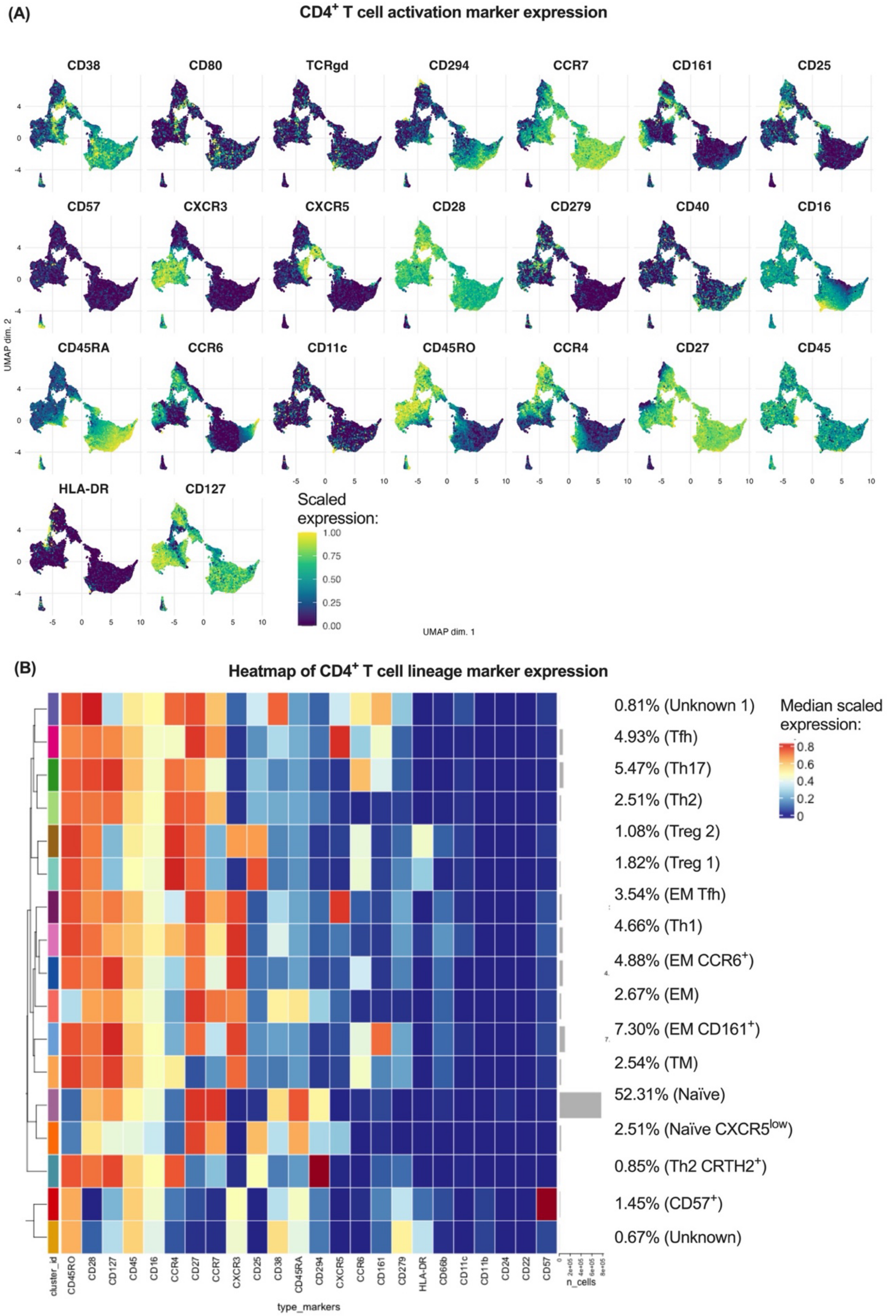
(A)
CD4+ T cell activation marker expression
CD38
CD80
TCRgd
CD294
CCR7
CD161
CD25
CD57
CXCR3
CXCR5
CD28
CD279
CD40
CD16
CD45RA
CCR6
CD11c
CD45RO
CCR4
CD27
CD45
HLA-DR
CD127
Scaled expression:
UMAP dim. 1
UMAP dim. 2
(B)
Heatmap of CD4+ T cell lineage marker expression
0.81% (Unknown 1)
4.93% (Tfh)
5.47% (Th17)
2.51% (Th2)
1.08% (Treg 2)
1.82% (Treg 1)
3.54% (EM Tfh)
4.66% (Th1)
4.88% (EM CCR6+)
2.67% (EM)
7.30% (EM CD161+)
2.54% (TM)
52.31% (Naïve)
2.51% (Naïve CXCR5low)
0.85% (Th2 CRTH2+)
1.45% (CD57+)
0.67% (Unknown)
Median scaled expression:
cluster_id
CD45RO
CD28
CD127
CD45
CD16
CCR4
CD27
CCR7
CXCR3
CD25
CD38
CD45RA
CD294
CXCR5
CCR6
CD161
CD279
HLA-DR
CD66b
CD11c
CD11b
CD24
CD22
CD57
n_cells
type_markers

(C) **CD8$^+$ T cell activation marker expression**

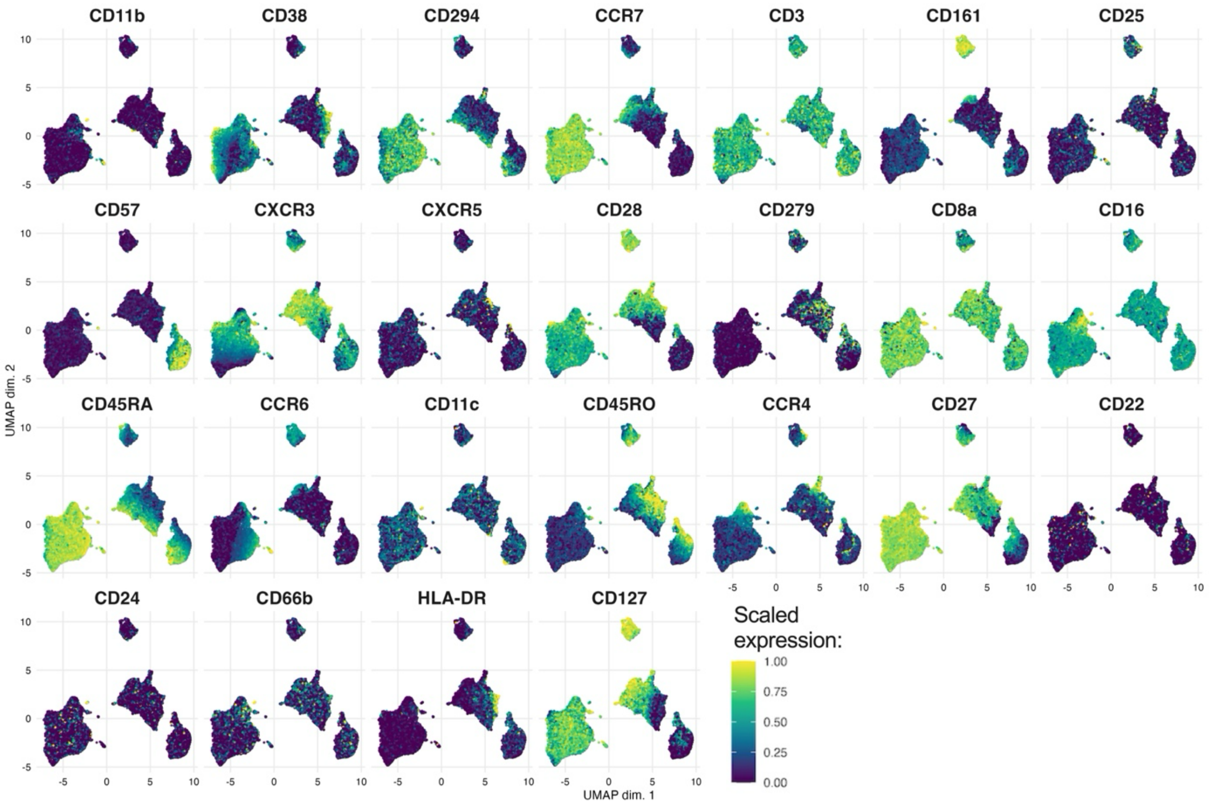

(D) **Heatmap of CD8$^+$ T cell lineage marker expression**

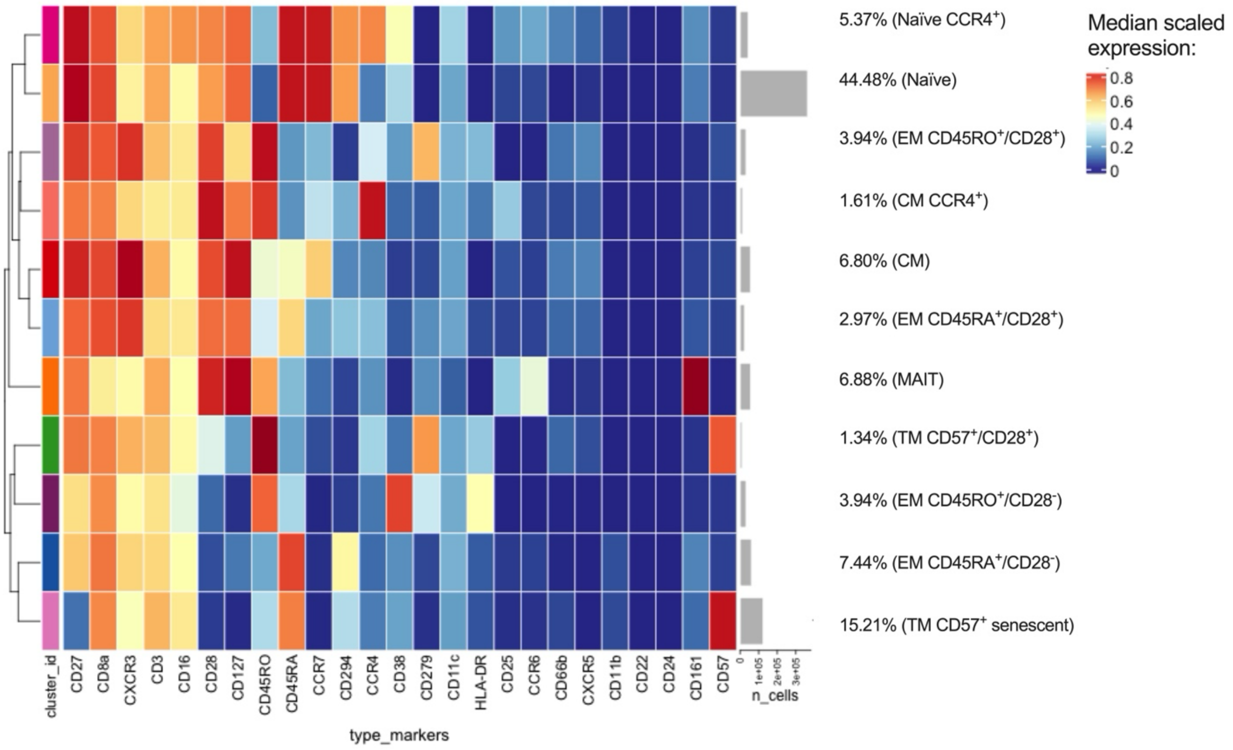

SUPPLEMENTARY FIGURE 5. Expression of state and type markers for identification of T cell subsets. Whole blood samples were pre-gated in FlowJo for quality control (n = 36), this analysis contains cells from this lineage: Cells/Intact cells/Single cells/Live cells/$CD45^+CD66b^-$/$CD14^-CD56^-$/$CD19^-CD3^+$. **(A)** State marker (variable) expression for $CD4^+$ T cells displayed by UMAP. **(B)** Type marker (stable) expression for $CD4^+$ T cells displayed by heatmap. Cell populations are reported as percentage of total $CD4^+$ cells from the UMAP. **(C)** State marker (variable) expression for $CD8^+$ T cells displayed by UMAP. **(D)** Type marker (stable) expression for $CD8^+$ T cells, displayed by heatmap. Cell populations are reported as percentage of total $CD8^+$ cells from the UMAP. TM = terminal memory, EM = effector memory, CM = central memory, Tfh = T follicular helper cells, Treg = regulatory T, MAIT = mucosal-associated invariant T cells.

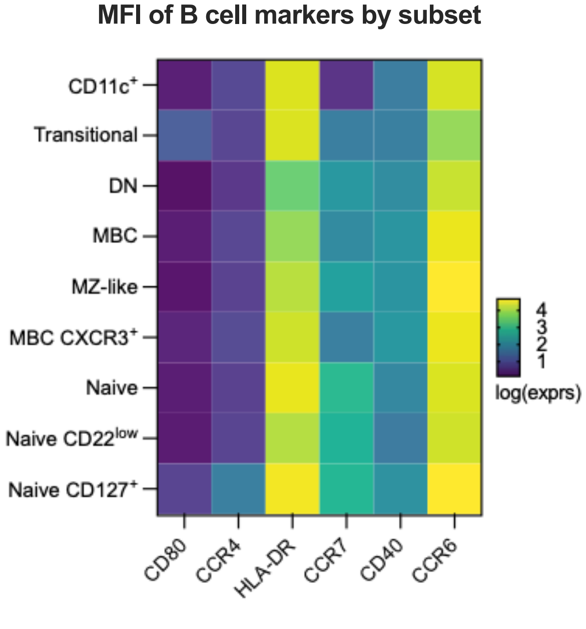

SUPPLEMENTARY FIGURE 6. MFI expression of relevant B cell markers for each subset. The MFI of relevant B cell markers was exported and compared between B cell subsets. Statistical significance of differences between groups was calculated using multiple non-parametric comparisons (Wilcoxon method). DN = double negative, MBC = memory B cell, MZ-like = marginal zone-like.

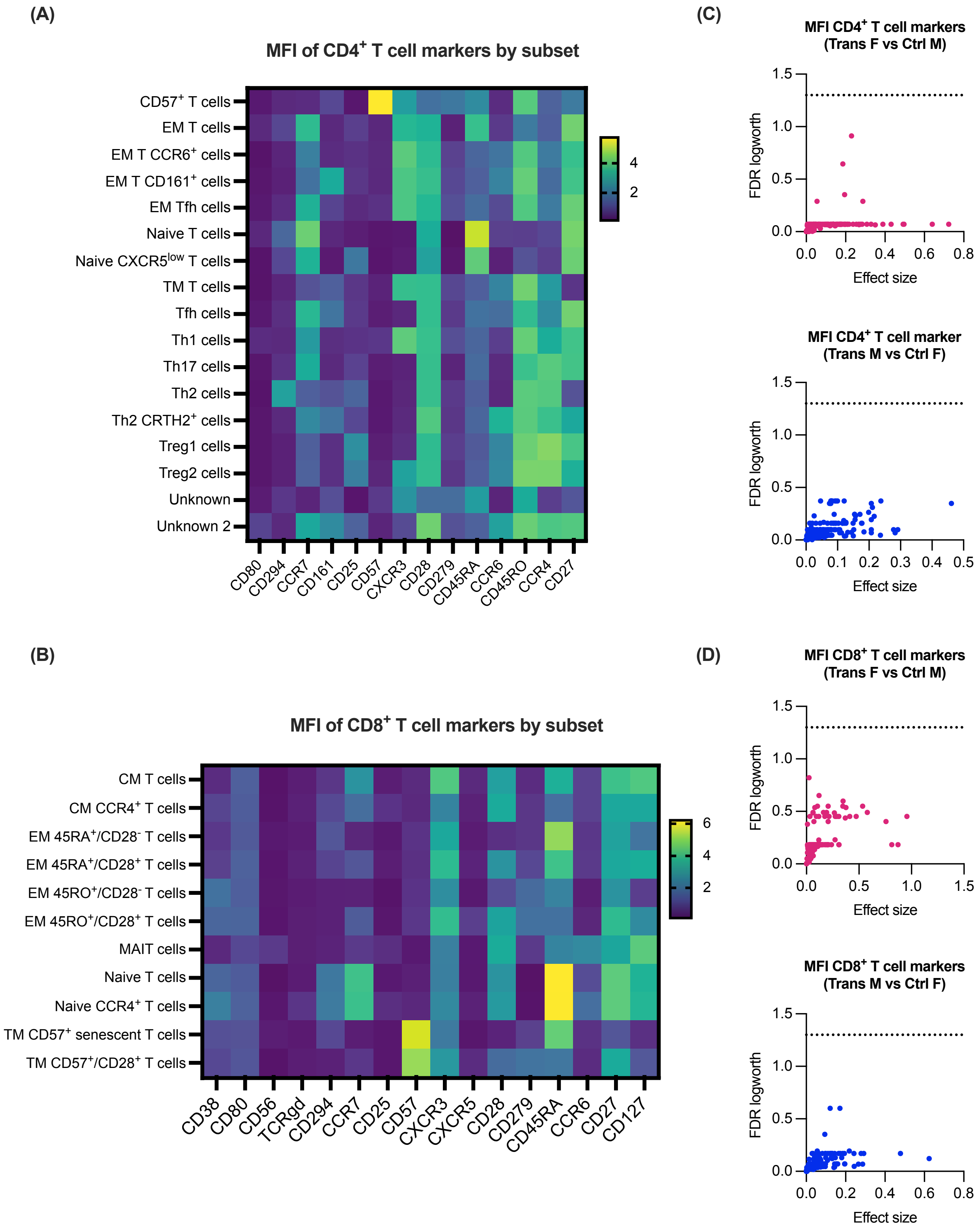

(A)
MFI of CD4+ T cell markers by subset
CD57+ T cells
EM T cells
EM T CCR6+ cells
EM T CD161+ cells
EM Tfh cells
Naive T cells
Naive CXCR5low T cells
TM T cells
Tfh cells
Th1 cells
Th17 cells
Th2 cells
Th2 CRTH2+ cells
Treg1 cells
Treg2 cells
Unknown
Unknown 2
CD80
CD294
CCR7
CD161
CD25
CD57
CXCR3
CD28
CD279
CD45RA
CCR6
CD45RO
CCR4
CD27
4
2
(B)
MFI of CD8+ T cell markers by subset
CM T cells
CM CCR4+ T cells
EM 45RA+/CD28- T cells
EM 45RA+/CD28+ T cells
EM 45RO+/CD28- T cells
EM 45RO+/CD28+ T cells
MAIT cells
Naive T cells
Naive CCR4+ T cells
TM CD57+ senescent T cells
TM CD57+/CD28+ T cells
CD38
CD80
CD56
TCRgd
CD294
CCR7
CD25
CD57
CXCR3
CXCR5
CD28
CD279
CD45RA
CCR6
CD27
CD127
6
4
2
(C)
MFI CD4+ T cell markers
(Trans F vs Ctrl M)
FDR logworth
Effect size
0.0
0.5
1.0
1.5
0.0
0.2
0.4
0.6
0.8
MFI CD4+ T cell marker
(Trans M vs Ctrl F)
FDR logworth
Effect size
0.0
0.5
1.0
1.5
0.0
0.1
0.2
0.3
0.4
0.5
(D)
MFI CD8+ T cell markers
(Trans F vs Ctrl M)
FDR logworth
Effect size
0.0
0.5
1.0
1.5
0.0
0.5
1.0
1.5
MFI CD8+ T cell markers
(Trans M vs Ctrl F)
FDR logworth
Effect size
0.0
0.5
1.0
1.5
0.0
0.2
0.4
0.6
0.8

SUPPLEMENTARY FIGURE 7. MFI of relevant T cell markers for each subset. (A, B) Heatmap of MFI of relevant T cell markers for $CD4^+$ T cell and $CD8^+$ T cell subsets. (**C, D)** Comparison of MFI of relevant markers for $CD8^+$ and $CD4^+$ T cell subsets between experimental groups. In A and B, statistical significance of differences between groups was calculated using multiple non-parametric comparisons (Wilcoxon method). In C and D, response screening of p values was performed using the false discovery rate (FDR) technique. Significance is indicated as * p<0.05, ** p<0.01, *** p<0.001. CM = central memory, EM = effector memory, Tfh = T follicular helper, TM = terminal memory, Treg = regulatory T.
